# Supplementary material for: Chemokines and galectins form heterodimers to modulate inflammation
Source: EMBO Rep. 2020 Feb 21;21(4):e47852. doi: 10.15252/embr.201947852 (PMC7132340; doi:10.15252/embr.201947852)
Supplement: Supplementary file 1 — Appendix [file EMBR-21-e47852-s001.pdf]

Appendix

A new encounter of mediators in inflammation: heterodimer formation between chemokines and galectins

Veit Eckardt,[1] Michelle C. Miller,[2] Xavier Blanchet,[1] Rundan Duan,[1] Julian Leberzammer,[1] Johan Duchene,[1] Oliver Soehnlein,[1] Remco T.A. Megens,[1] Anna-Kristin Ludwig,[3] Aurelio Dregni,[2] Alexander Faussner,[1] Kanin Wichapong,[4] Hans Ippel,[4] Ingrid Dijkgraaf,[4] Herbert Kaltner,[5] Yvonne Doring,[1] Kiril Bidzhekov,[1] Tilman M. Hackeng,[4] Christian Weber,[1,4,5] Hans-Joachim Gabius,[3] Philipp von Hundelshausen,[1,5,*] and Kevin H. Mayo[2,*]

This PDF file includes:

Appendix Figure S1. CXCL12/Gal-3 heterodimer formation in the presence of lactose and heparin. p. 2

Appendix Figure S2. Co-expression of Gal-3 and chemokines in mouse tissue. p. 3

Appendix Figure S3. HSQC spectra of CXCL12 and Gal-3 CRD. pp. 4-5

Appendix Figure S4. Cross-linking of CXCL12 and Gal-3 CRD. p. 6

Appendix Figure S5. CXCL12-induced chemical shifts of full-length ^{15}N-labeled Gal-3. p. 7

Appendix Figure S6. MD-based energy-minimized structure of the CXCL12/Gal-3 CRD heterodimer. pp. 8-9

Appendix Figure S7. Binding of CXCL12 to Gal-3, Gal-3 CRD and their mutants. p. 10

Appendix Figure S8. Binding kinetics of Gal-3 mutants to CXCL12 and ASF. pp. 11-12

Appendix Figure S9. Effect of DMJ, galectin-mediated glycan binding and NUCC-390 on the chemotaxis of Jurkat T cells. p. 13

Appendix Figure S10. Analysis of leukocyte subsets from the peritoneal lavage of mice. p. 14

Appendix Table S1. ΔG of heterodimer formation between CXCL12 and Gal-3 CRD and its mutants. p. 15

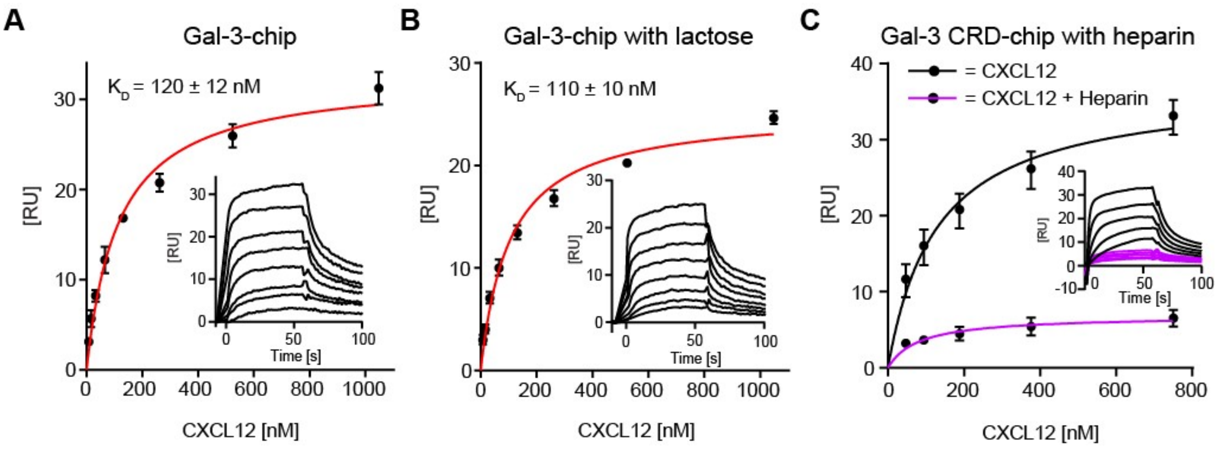

Appendix Figure S1. CXCL12/Gal-3 heterodimer formation in the presence of lactose and heparin.

A-B Gal-3 was immobilized using thiol-coupling on a C1-sensor chip to a density of 650 RU and serial dilutions of CXCL12 were passed over the chip either in the (A) absence or (B) presence of 70 mM lactose (n=3). The K_D values were obtained by fitting the signals of the steady-state phases against the concentration of the chemokine (red) using a single-site model. A representative sensorgram is provided in the inset to each panel.

C For kinetic analysis CXCL12 at increasing concentrations alone or together with heparin (0.5 µg/mL) was passed over the Gal-3 CRD chip (CXCL12 alone in black, and with heparin in purple).

Data information: Insets show exemplary sensorgrams of CXCL12 on immobilized galectin. Data represent the mean ± SD from three independent experiments.

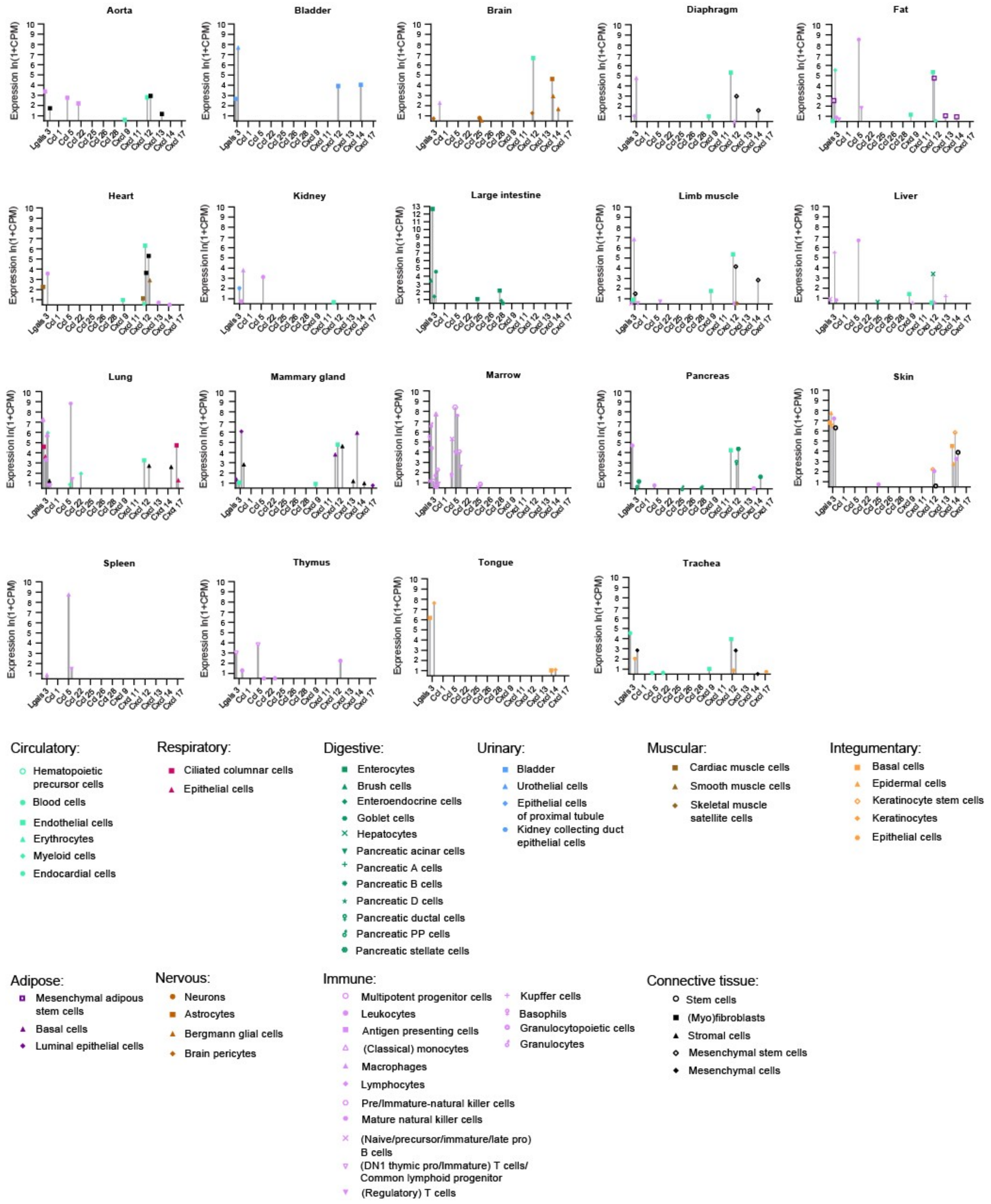

Appendix Figure S2. Co-expression of Gal-3 and chemokines in mouse tissue.
Gene expression data from FACS-based full-length transcript analyses were obtained from *Tabula Muris*, a compendium of single cell transcriptome data derived from mouse organs [36]. The mean gene count expressed as natural logarithm of copies per million (CPM) is shown.

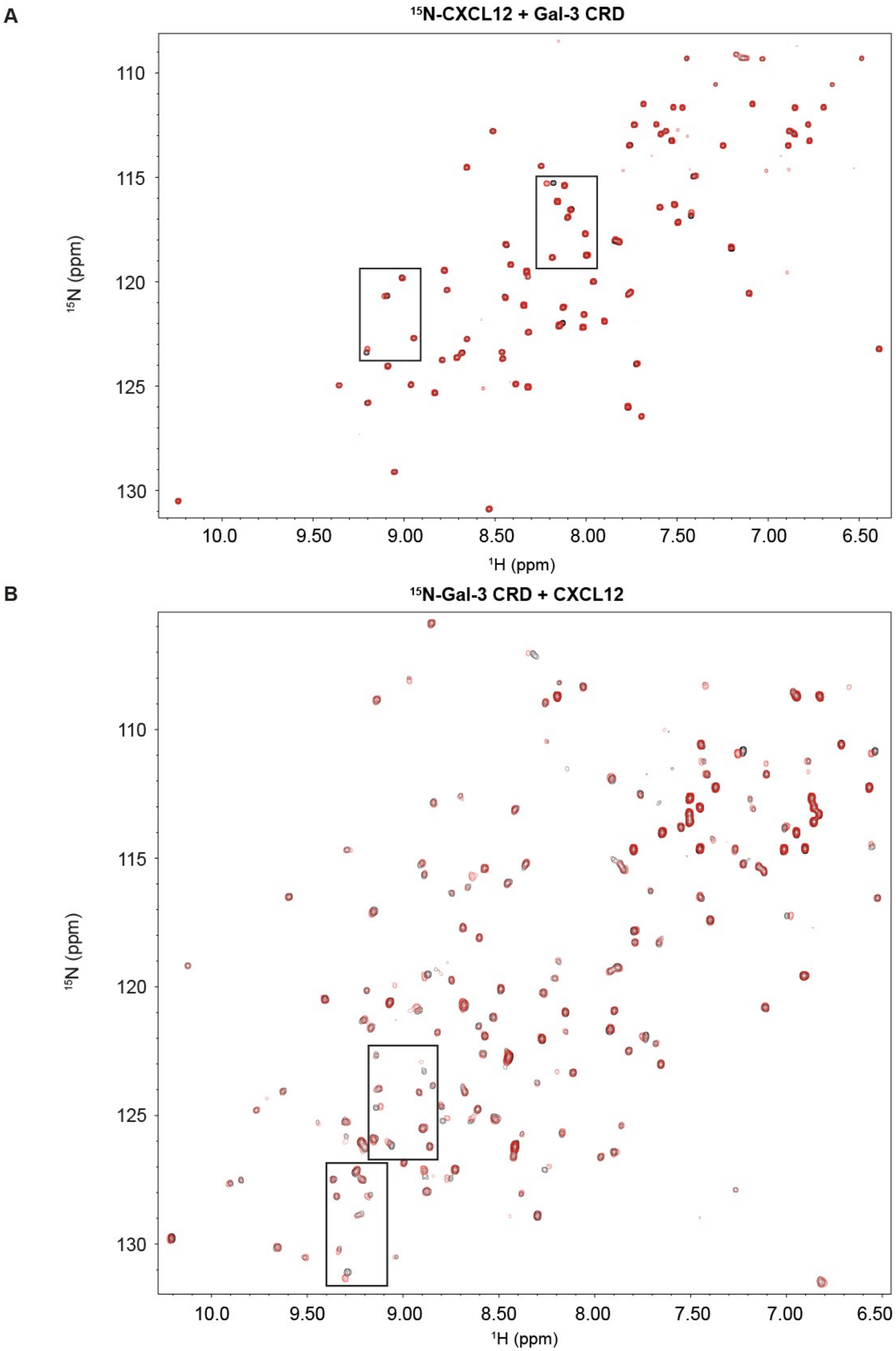
A
^{15}N-CXCL12 + Gal-3 CRD
^{15}N (ppm)
^{1}H (ppm)
B
^{15}N-Gal-3 CRD + CXCL12
^{15}N (ppm)
^{1}H (ppm)

Appendix Figure S3. ^{1}H-^{15}N HSQC spectra of CXCL12 and Gal-3 CRD.

A-B Full ^{1}H-^{15}N HSQC spectra are overlaid depicting (A) 10 μM ^{15}N-enriched CXCL12 alone (black) and in the presence of 330 μM unlabeled Gal-3 CRD (red) and (B) 30 μM ^{15}N-enriched Gal-3 CRD alone (black) and in the presence of 500 μM unlabeled CXCL12 (red). The boxed regions in these spectra are shown in greater detail in Figure 2A and B.

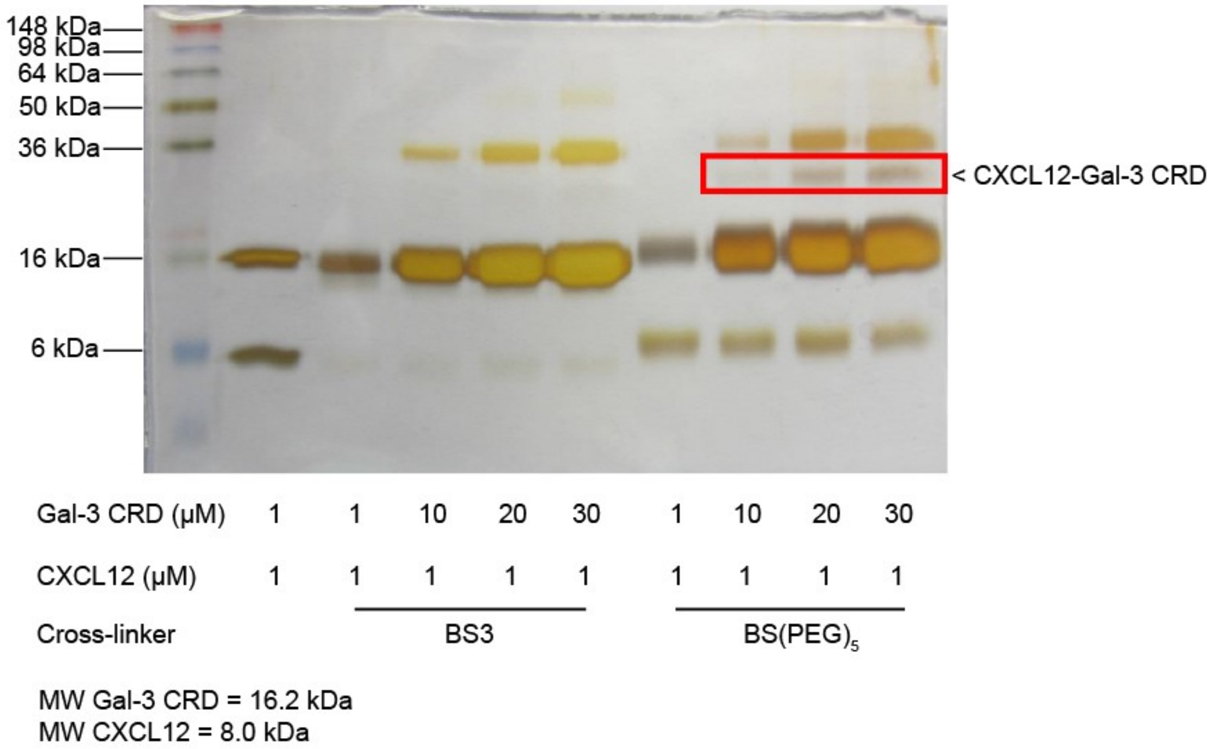

Appendix Figure S4. Cross-linking of CXCL12 and Gal-3 CRD.

CXCL12 and Gal-3 CRD were incubated with cross-linkers BS3 and BS(PEG)$_5$. SDS PAGE and silver staining of the gel were performed. Using increasing concentrations of Gal-3 CRD and the longer BS(PEG)$_5$ linker, bands appeared at the position expected for a molecular weight consistent with that of the CXCL12/Gal-3 CRD heterodimer (red rectangle). This is a representative image of three independent experiments.

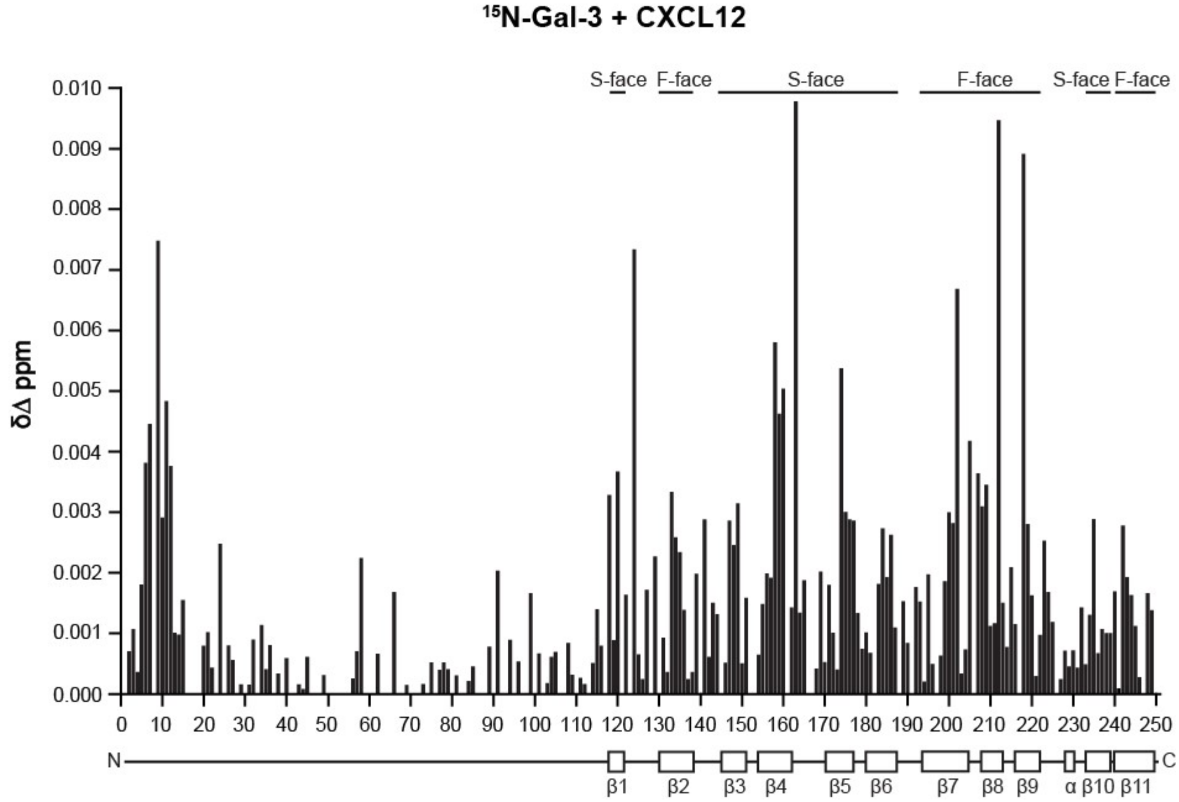

Appendix Figure S5. CXCL12-induced chemical shifts of full-length ^{15}N-labeled Gal-3.

Δδ Values plotted vs. the amino acid sequence and secondary structure of Gal-3 are shown for 30 µM ^{15}N-enriched Gal-3 in the presence of 500 µM unlabeled CXCL12.

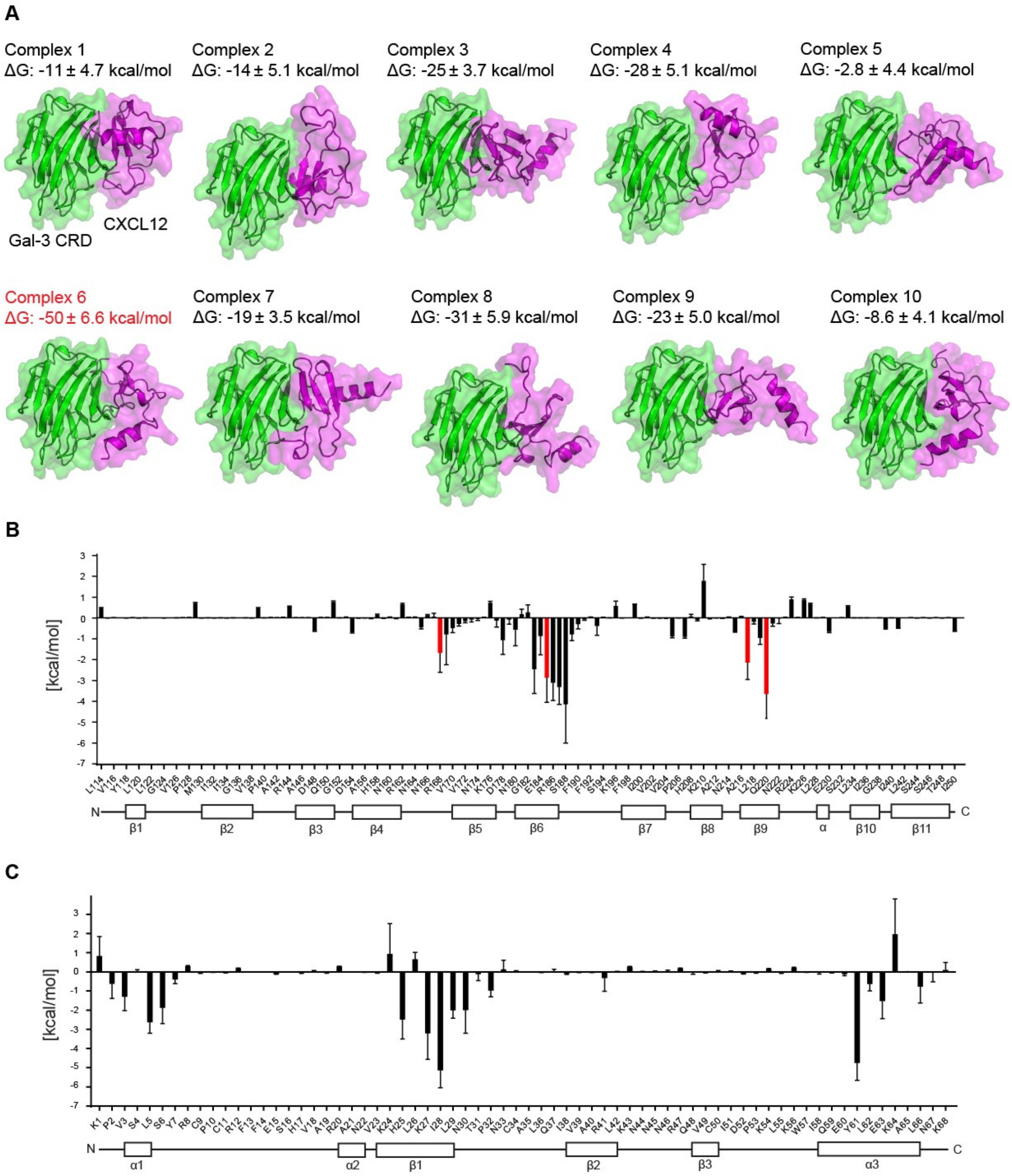
A
Complex 1
ΔG: -11 ± 4.7 kcal/mol
Complex 2
ΔG: -14 ± 5.1 kcal/mol
Complex 3
ΔG: -25 ± 3.7 kcal/mol
Complex 4
ΔG: -28 ± 5.1 kcal/mol
Complex 5
ΔG: -2.8 ± 4.4 kcal/mol
Gal-3 CRD
CXCL12
Complex 6
ΔG: -50 ± 6.6 kcal/mol
Complex 7
ΔG: -19 ± 3.5 kcal/mol
Complex 8
ΔG: -31 ± 5.9 kcal/mol
Complex 9
ΔG: -23 ± 5.0 kcal/mol
Complex 10
ΔG: -8.6 ± 4.1 kcal/mol
B
[kcal/mol]
N
C
β1
β2
β3
β4
β5
β6
β7
β8
β9
α
β10
β11
C
[kcal/mol]
N
C
α1
α2
β1
β2
β3
α3

Appendix Figure S6. MD-based energy-minimized structure of the CXCL12/Gal-3 CRD heterodimer.

A 10 Potential structures of the CXCL12/Gal-3 CRD heterodimer consistent with the NMR results (Figure 2A-D, Appendix Figure S3A and B) were modeled and the ΔG values of interaction were calculated. Complex 6 is the thermodynamically most favorable conformation, because its formation generates the largest ΔG value, thus the highest affinity (red).

B-C Decomposition analyses of all residues of (B) Gal-3 CRD and (C) CXCL12 in a CXCL12/Gal-3 CRD heterodimer were performed. Secondary structures are shown below the graph. Sites of amino acid substitutions are highlighted in red.

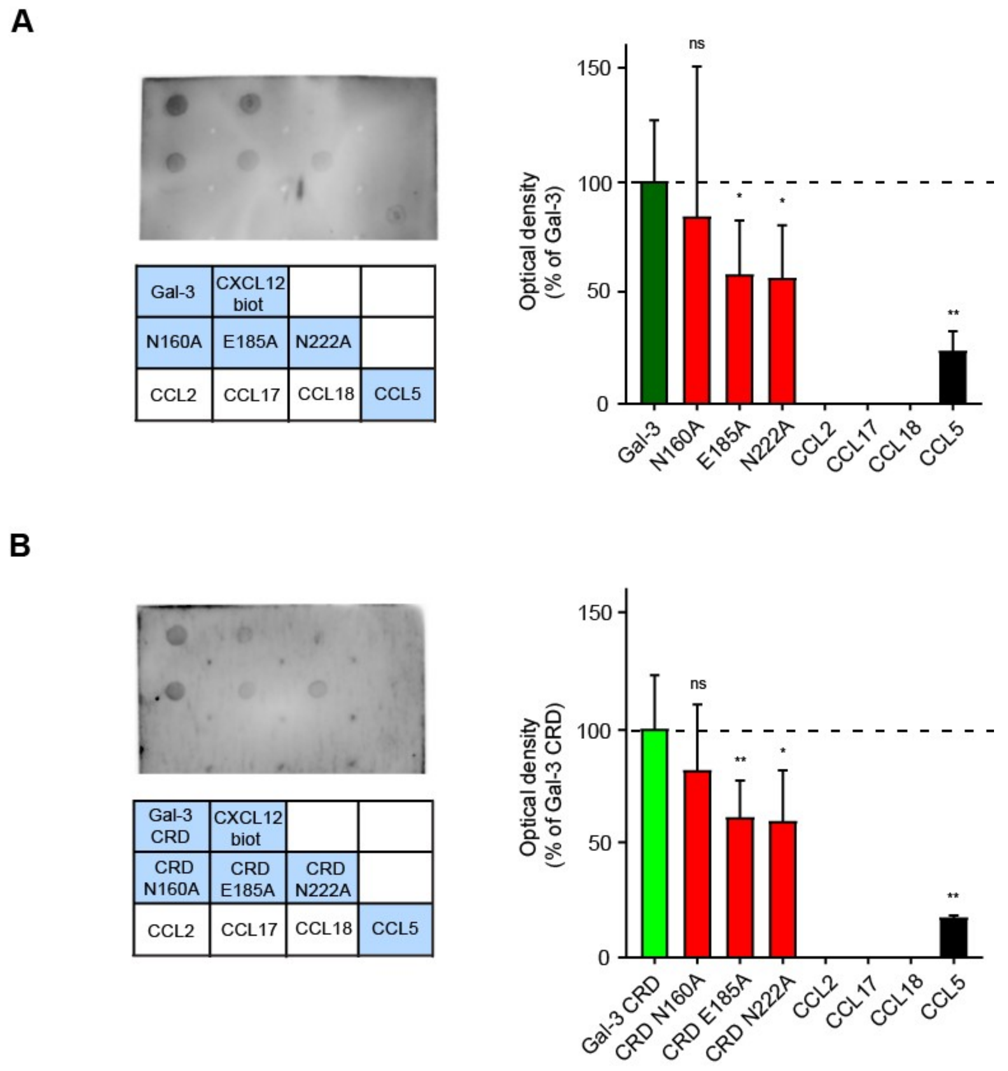

Appendix Figure S7. Binding of CXCL12 to Gal-3, Gal-3 CRD and their mutants.

A-B Membrane blot experiments were performed with (A) Gal-3 and (B) Gal-3 CRD and their mutants immobilized on the membrane and biotinylated CXCL12 in solution, as exemplified on the left in each panel. Chemokines served as negative and positive controls as described [15]. Membranes were subjected to densitometric analysis with values normalized to the galectin, as shown on the right (results of WT Gal-3 and Gal-3 CRD in green and light green respectively, mutants in red).

Data information: Data represent the mean ± SD from three independent experiments and were statistically analyzed against the effect of the galectin by using an unpaired t-test (*=P≤0.05, **=P≤0.01).

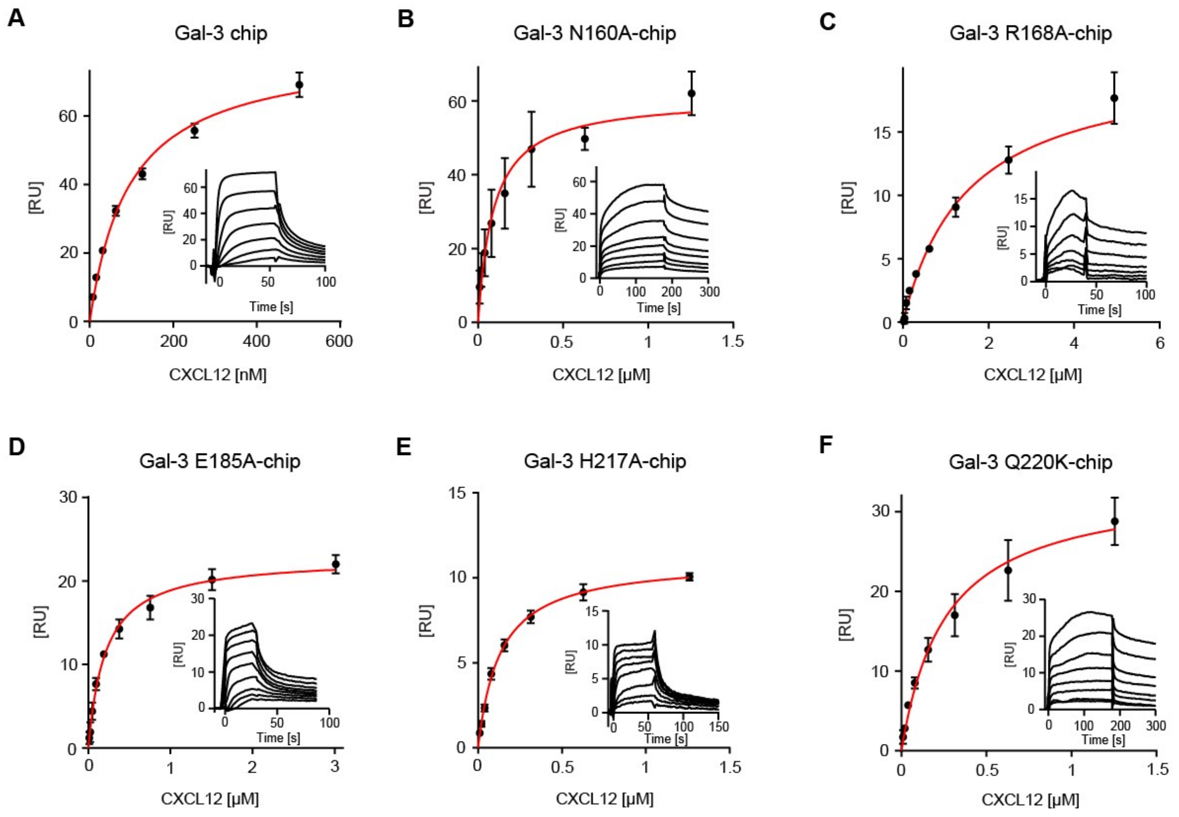

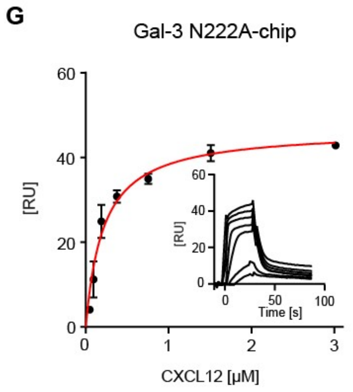

H

Mutant	K_D [nM]
**Gal-3**	**96 ± 6.9**
Gal-3 N160A	91 ± 20
Gal-3 R168A	1500 ± 150
Gal-3 E185A	210 ± 16
Gal-3 H217A	130 ± 6.9
Gal-3 Q220K	260 ± 37
Gal-3 N222A	210 ± 34

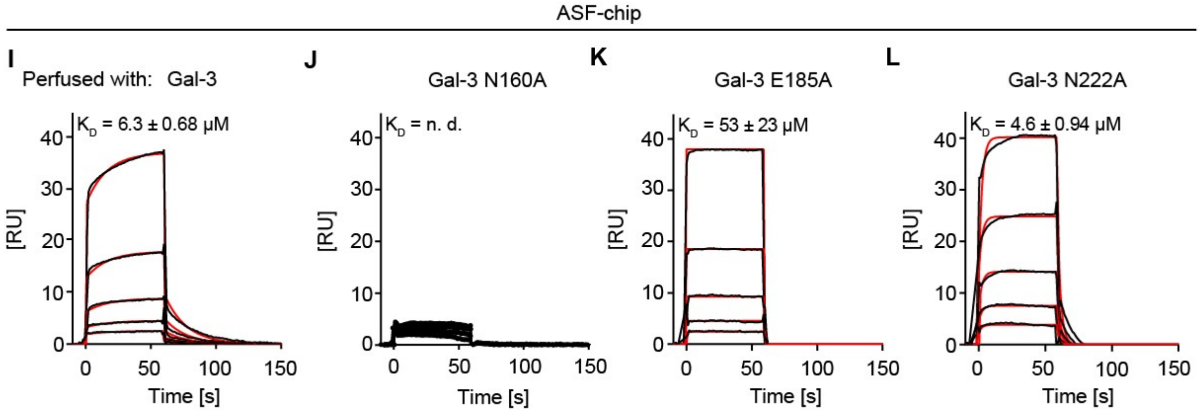

Appendix Figure S8. Binding kinetics of Gal-3 mutants to CXCL12 and ASF.

A-G For kinetic SPR analysis (A) Gal-3 (density 239 RU), (B) Gal-3 N160A (density 291 RU), (C) Gal-3 R168A (density 160 RU), (D) Gal-3 E185A (density 264 RU), (E) Gal-3 H217A (density 197 RU), (F) Gal-3 Q220K (density 216 RU) and (G) Gal-3 N222A (density 258 RU) were immobilized on sensor chips and increasing concentrations of CXCL12 were passed over the flow cell (A-G: n = 3).

H K_D values were determined by fitting the signals of steady-state phases vs. the concentration of CXCL12 (A-G in red).

I-L The interaction of Gal-3 mutants with glycans was assessed by determining the binding capacity of increasing concentrations of galectin to ASF immobilized at a density of 460 RU on the sensor chip surface (representative example of n = 3). K_D values were obtained by fitting the graphs according to a 1:1 Langmuir interaction model (in red).

Data information: Data represent the mean ± SD from three independent experiments.

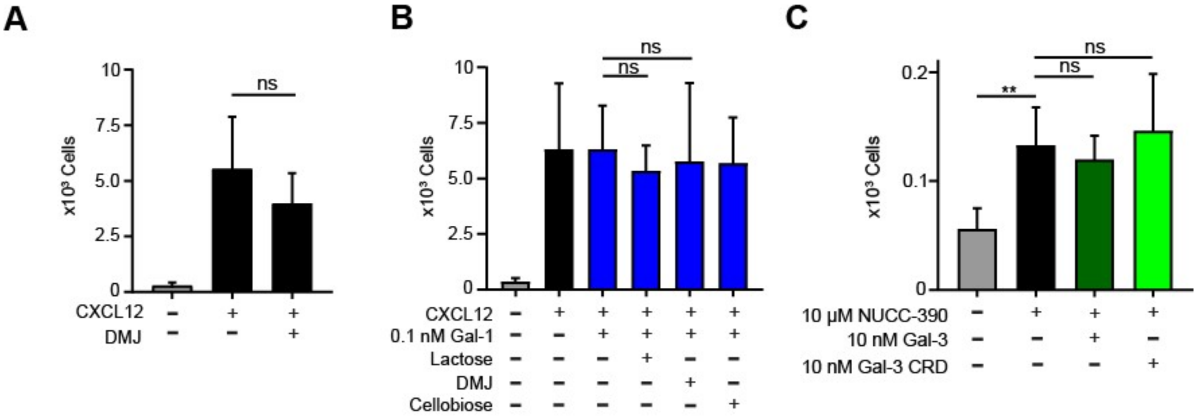

Appendix Figure S9. Effect of DMJ, galectin-mediated glycan binding and NUCC-390 on the chemotaxis of Jurkat T cells.

A Jurkat T cells migrated to 10 nM CXCL12 alone with and without pretreatment of cells with 150 µM DMJ, an inhibitor of N-linked glycosylation, for 24 hours (n = 3).

B Jurkat T cells were allowed to migrate in the presence of 10 nM CXCL12 alone or in combination with 0.1 nM Gal-1, 70 mM lactose, DMJ and 70 mM cellobiose (n = 3).

C Jurkat T cells migrated with 10 µM NUCC-390 (a CXCR4 agonist) and with 10 nM Gal-3 and Gal-3 CRD (n = 3).

Data information: Cell migration is shown as absolute cell count. Data represent the mean ± SD from three independent experiments and were statistically analyzed by using an unpaired t-test as indicated (**=p≤0.01).

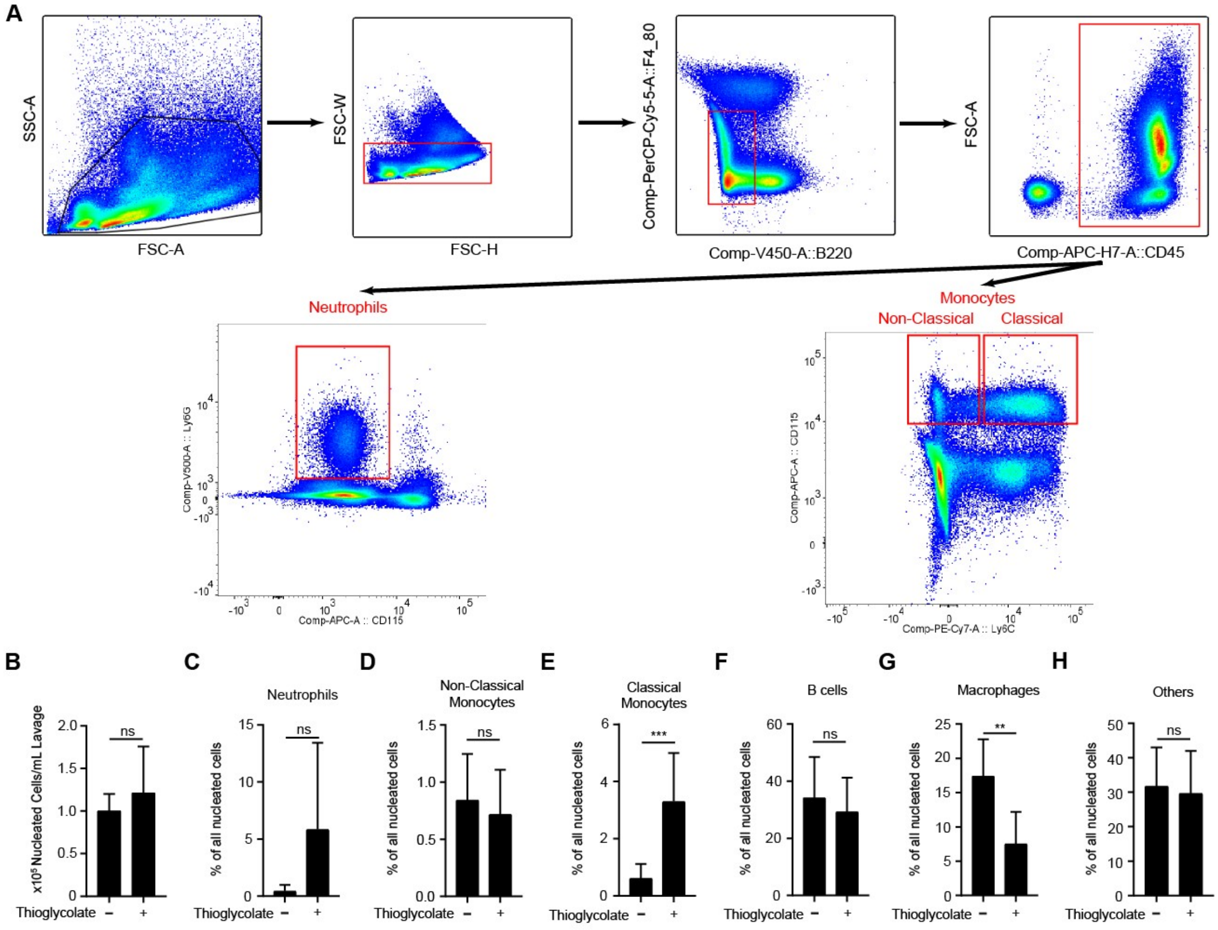

Appendix Figure S10. Analysis of leukocyte subsets from the peritoneal lavage of mice.

A Sample data from a wild type mouse 18 h after injection of 4% thioglycolate are depicted. Leukocyte singlets were defined as FSC-H high and FSC-W low. Neutrophils, non-classical monocytes and classical monocytes were defined as B220-/F4-80-/CD45+/CD115-/Ly6G+, B220-/F4-80-/CD45+/CD115+/Ly6Clo and B220-/F4-80-/CD45+/CD115+/Ly6Chi respectively.

B-H (B) All leukocytes in the peritoneal lavage and (C-H) percentages of leukocyte subsets of WT mice 18 h after injection with PBS and TG are shown (PBS: n = 6, TG: n = 10).

Mutant	ΔG [kcal/mol]
**Gal-3 WT**	**-50 ± 6.6**
**N160A**	**-48 ± 6.4**
N160D	-43 ± 8.8
**R168A**	**-33 ± 4.3**
R169A	-34 ± 7.0
R183A	-34 ± 5.4
R183D	-51 ± 7.7
R183E	-42 ± 5.8
E184A	-33 ± 5.5
E184N	-42 ± 7.7
E184Q	-40 ± 6.5
**E185A**	**-35 ± 5.7**
S188A	-34 ± 7.5
K210A	-53 ± 5.6
K210D	-57 ± 6.8

Mutant	ΔG [kcal/mol]
K210E	-50 ± 6.5
**H217A**	**-38 ± 6.9**
H217F	-26 ± 5.6
H217Y	-49 ± 6.4
Q220A	-37 ± 4.4
Q220D	-55 ± 9.5
**Q220E**	**-58 ± 5.4**
**Q220K**	**-31 ± 5.8**
Q220R	-30 ± 4.4
**N222A**	**-44 ± 7.1**
N222D	-37 ± 4.1
**N222E**	**-27 ± 4.7**
R224A	-48 ± 6.8
R224D	-35 ± 5.0
R224E	-27 ± 5.8

Appendix Table S1. ΔG of the heterodimer between CXCL12 and Gal-3 CRD and its mutants.

Data from computational analyses of ΔG generated by CXCL12/Gal-3 CRD heterodimerization for WT and mutated Gal-3 CRD are provided. Mutants that were experimentally tested are labeled in color based on ΔG values compared to WT (see also Appendix Fig S6B and C).
