# Supplementary material for: Chemokines and galectins form heterodimers to modulate inflammation
Source: EMBO Rep. 2020 Feb 21;21(4):e47852. doi: 10.15252/embr.201947852 (PMC7132340; doi:10.15252/embr.201947852)
Supplement: Supplementary file 2 — Expanded View Figures PDF [file EMBR-21-e47852-s002.pdf]

# Expanded View Figures

**Figure EV1. Physical interaction of Gal-3 and Gal-1 with CC and CXC chemokines.**

A–D Chemokines were immobilized on a nitrocellulose membrane and incubated with (A) TBS or (B) TBS containing biotinylated Gal-1. The membranes were then stepwise incubated in solutions with SA-HRP and chemiluminescence reagents. (C, D) The blots were subjected to densitometric analysis.

E–G Binding of galectins to chemokines was further assessed by immobilizing (E) Gal-3, (F) Gal-3 CRD, and (G) Gal-1 on sensor chips at a density of 700 RU and detecting signals of human chemokines under flow. Signals of the solid-phase assays (Fig 1E) are depicted in light blue ($n$ = 3). Data represent mean ± SD from three independent experiments.


© 2020 The Authors

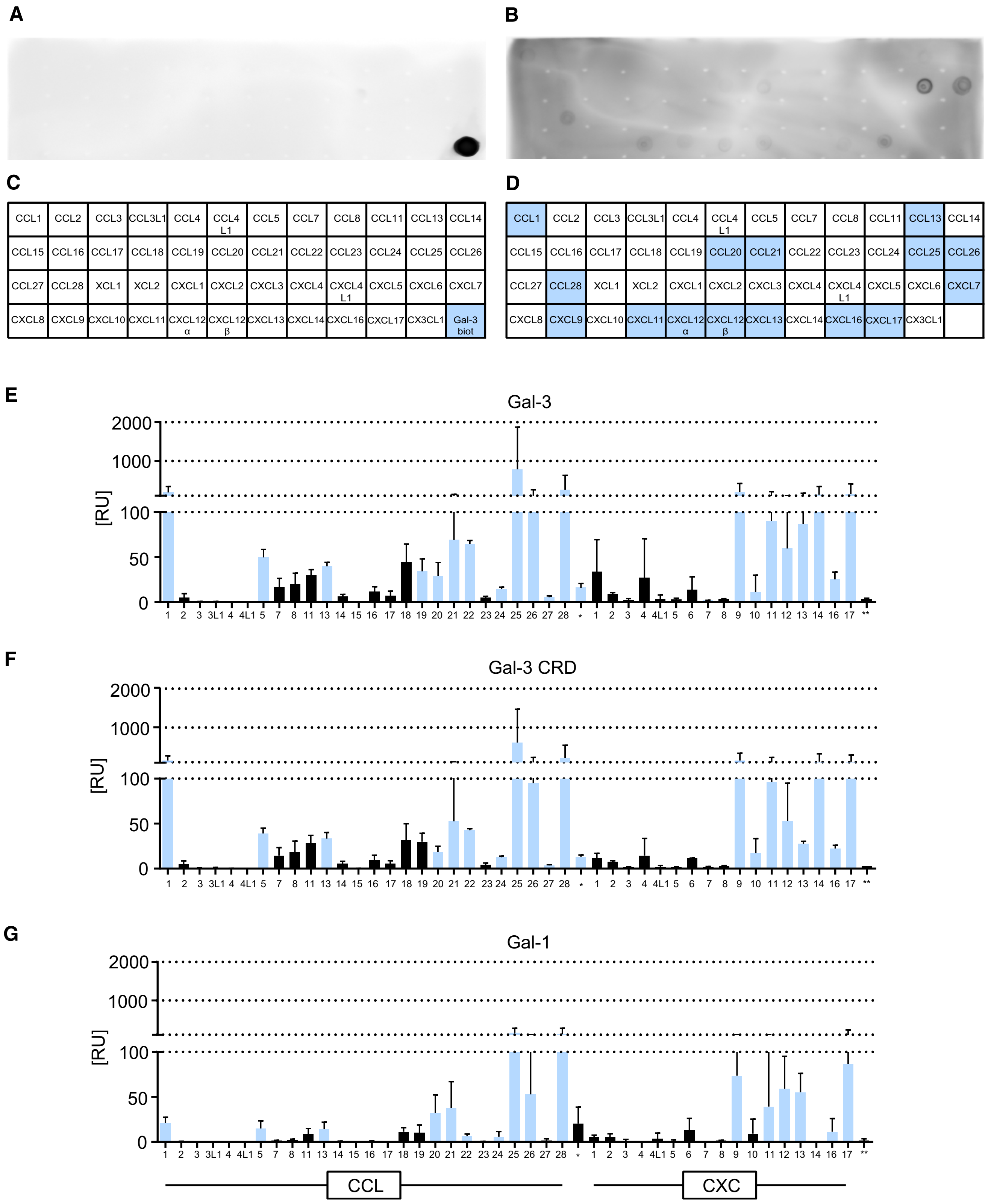
A
B
C
CCL1 CCL2 CCL3 CCL3L1 CCL4 CCL4 L1 CCL5 CCL7 CCL8 CCL11 CCL13 CCL14
CCL15 CCL16 CCL17 CCL18 CCL19 CCL20 CCL21 CCL22 CCL23 CCL24 CCL25 CCL26
CCL27 CCL28 XCL1 XCL2 CXCL1 CXCL2 CXCL3 CXCL4 CXCL4 L1 CXCL5 CXCL6 CXCL7
CXCL8 CXCL9 CXCL10 CXCL11 CXCL12 α CXCL12 β CXCL13 CXCL14 CXCL16 CXCL17 CX3CL1 Gal-3 biot
D
CCL1 CCL2 CCL3 CCL3L1 CCL4 CCL4 L1 CCL5 CCL7 CCL8 CCL11 CCL13 CCL14
CCL15 CCL16 CCL17 CCL18 CCL19 CCL20 CCL21 CCL22 CCL23 CCL24 CCL25 CCL26
CCL27 CCL28 XCL1 XCL2 CXCL1 CXCL2 CXCL3 CXCL4 CXCL4 L1 CXCL5 CXCL6 CXCL7
CXCL8 CXCL9 CXCL10 CXCL11 CXCL12 α CXCL12 β CXCL13 CXCL14 CXCL16 CXCL17 CX3CL1
E
Gal-3
F
Gal-3 CRD
G
Gal-1
[RU]
2000
1000
100
50
0
1 2 3 3L1 4 4L1 5 7 8 11 13 14 15 16 17 18 19 20 21 22 23 24 25 26 27 28 * 1 2 3 4 4L1 5 6 7 8 9 10 11 12 13 14 16 17 **
CCL
CXC

**Figure EV1.**

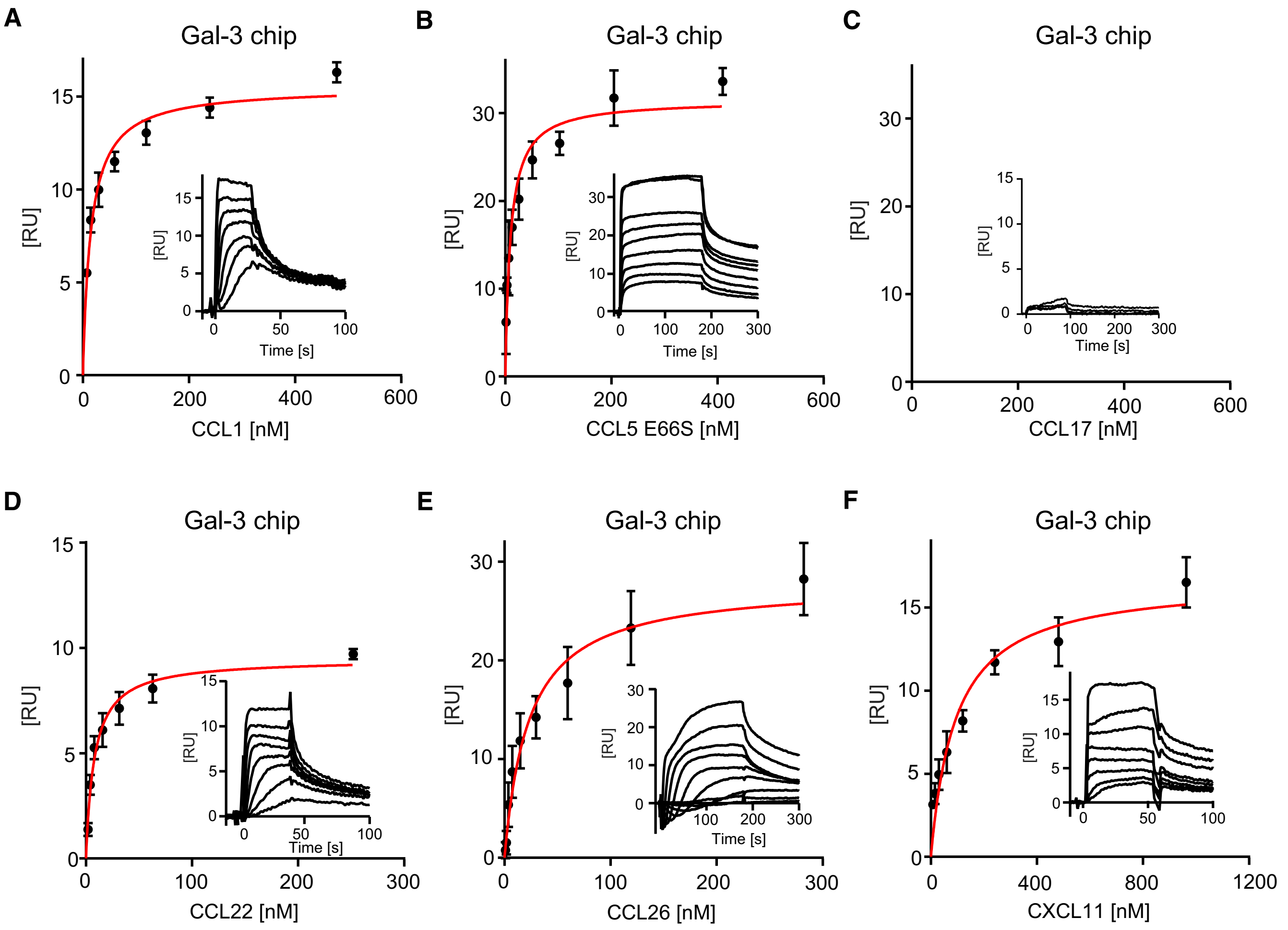


**G**

| Chemokine | $K_D$ [nM] |
|---|---|
| CCL1 | 15 ± 1.7 |
| CCL5 E66S | 99 ± 16 |
| CCL17 | --- |
| CCL22 | 7.9 ± 1.0 |
| CCL26 | 23 ± 4.8 |
| CXCL11 | 95 ± 17 |

**Figure EV2. Heterodimer formation between Gal-3 and chemokines.**

A–F For kinetic SPR analyses, Gal-3 was immobilized on sensor chips to a density of 650 RU, and increasing concentrations of (A) CCL1, (B) CCL5E66S, (C) CCL17, (D) CCL22, (E) CCL26, and (F) CXCL11 were passed over the flow cell. Insets show representative sensorgrams of chemokines on Gal-3. Data represent the mean ± SD of three independent experiments.

G $K_D$ values were calculated by fitting signals of steady-state phases vs. the concentration of the chemokine (A–F in red).


© 2020 The Authors

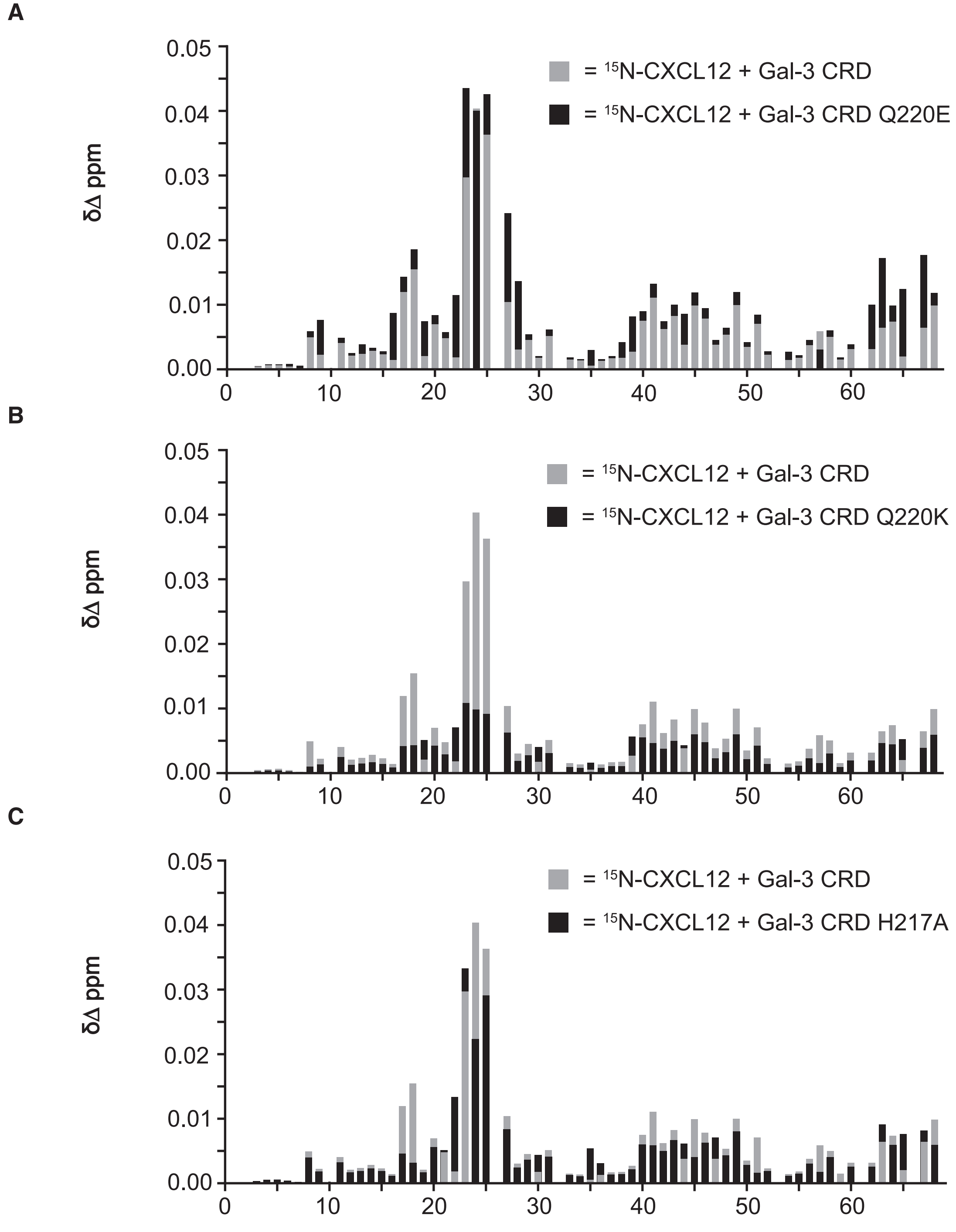


**Figure EV3. $^{1}H$–$^{15}N$ chemical shift maps for $^{15}N$-labeled CXCL12 with WT Gal-3 CRD and its mutants.**

A–C Δδ Values plotted vs. the amino acid sequence of CXCL12 are shown for 30 μM $^{15}N$-enriched CXCL12 in the presence of 500 μM label-free Gal-3 CRD mutants (A) Q220E, (B) Q220K, and (C) H217A (black). The results of the experiment with WT Gal-3 CRD from Fig 2A are overlaid in gray.

© 2020 The Authors

**Figure EV4. Co-localization of CXCL12 and Gal-3 on cells *in vivo*.**

A, B Proximity of Gal-3 and CXCL12 on cells from peritoneal lavages after injection of (A) PBS or (B) TG was determined by PLA (representative example of $n = 3$). White scale bar: 10 μm.

C, D Co-localization of Gal-3 and CXCL12 in frozen sections of lymph nodes from (C) WT and (D) CXCL12$^{-/-}$ mice was determined by immunofluorescence staining using antibodies against Gal-3 and CXCL12 (representative example of $n = 3$). Scale bar: 40 μm. Arrows indicate subcapsular sinus macrophages (SSM).

E Co-localization on the same sections was also detected by PLA (representative example of $n = 3$). Scale bar: 40 μm.


© 2020 The Authors

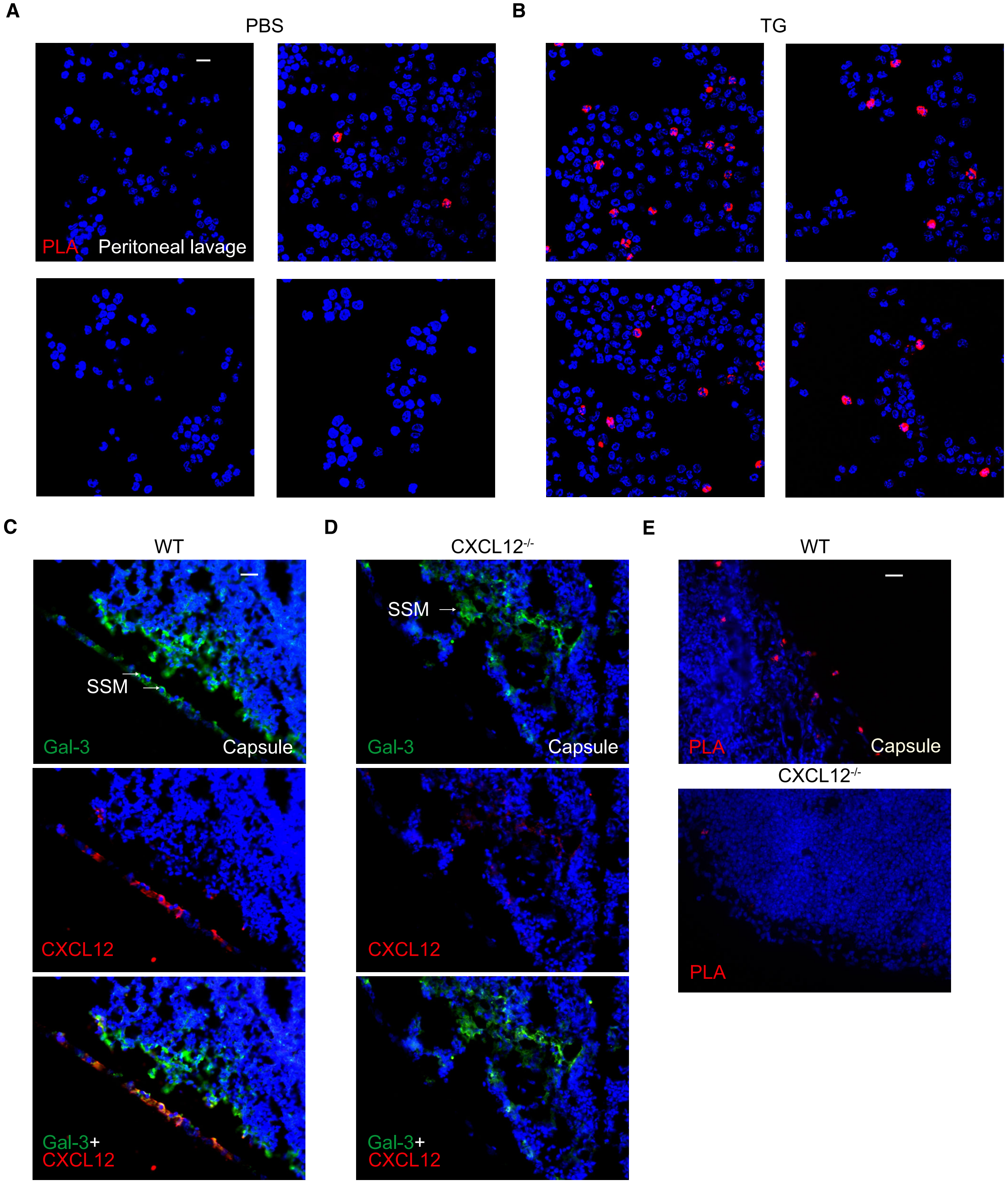
A
PBS
PLA
Peritoneal lavage
B
TG
C
WT
SSM
Gal-3
Capsule
CXCL12
Gal-3+
CXCL12
D
CXCL12-/-
SSM
Gal-3
Capsule
CXCL12
Gal-3+
CXCL12
E
WT
PLA
Capsule
CXCL12-/-
PLA

**Figure EV4.**

**Figure EV5. MD simulation of a ternary complex between CXCL12, Gal-3 CRD, and CXCR4 in a lipid bilayer environment.**

Complex 6 was superimposed onto the viral chemokine antagonist vMIP-II in complex with CXCR4 (PDB access code 4RWS). Subsequently, the viral chemokine antagonist vMIP-II was deleted, and the complex between monomeric CXCR4 in complex with the CXCL12/Gal-3 CRD heterodimer was obtained. This model was refined by energy minimization in the course of MD simulations (50 ns) with coordinates and orientation from another monomer of CXCR4 (CXCR4 as dimer (PDB access code 3ODU), monomers in cyan and yellow, CXCL12 in magenta, and Gal-3 CRD in light green). The inset shows that N180 (Gal-3 CRD) forms H-bonds (dashed red lines) with N97 (CXCR4) and R183 (Gal-3 CRD) can do so with E31 (CXCR4) and with R8 (CXCL12).

 © 2020 The Authors

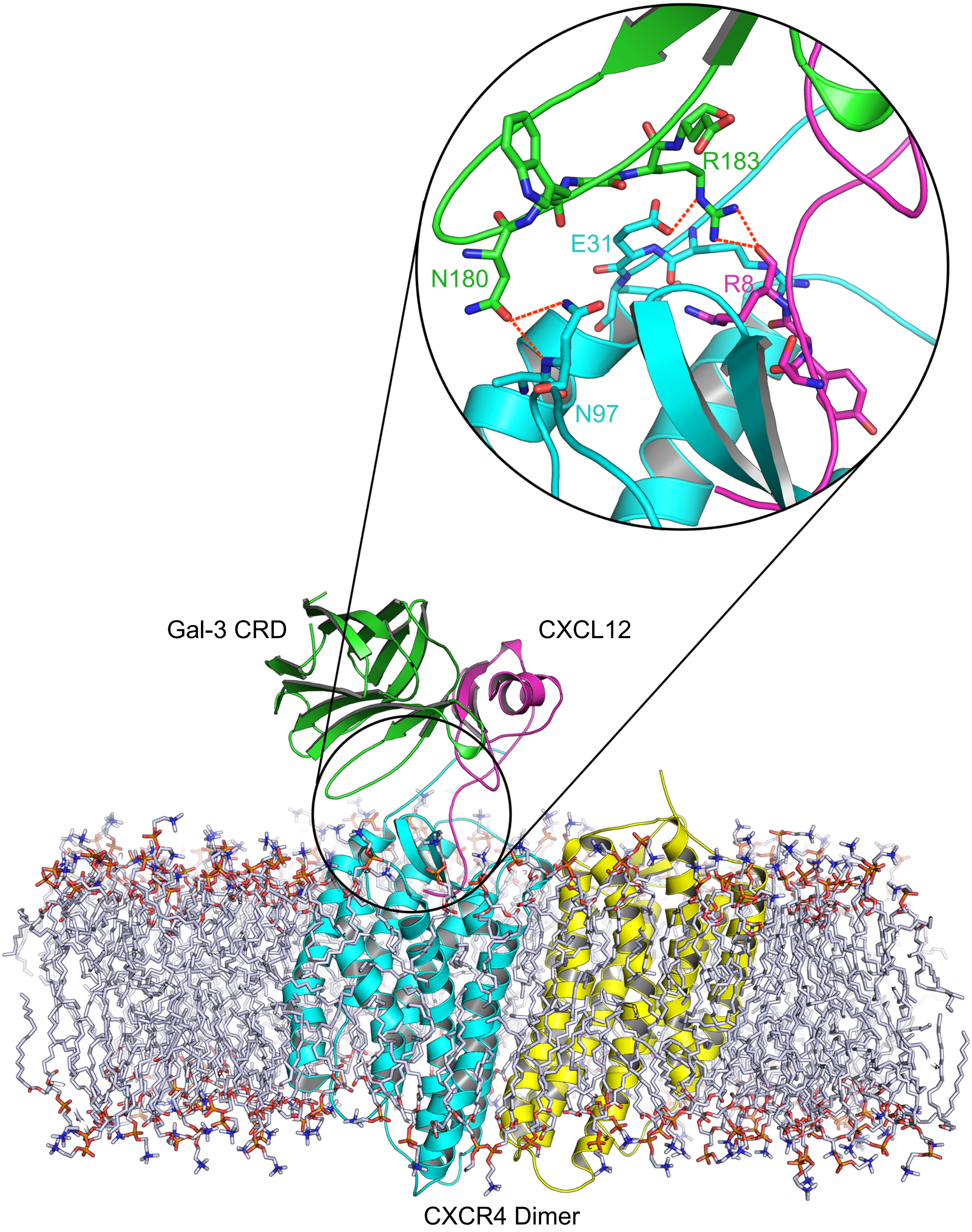


**Figure EV5.**

© 2020 The Authors
